# Effect of storage time of paraffin sections on the expression of PD-L1 (SP142) in invasive breast cancer

**DOI:** 10.1186/s13000-023-01423-8

**Published:** 2023-12-05

**Authors:** Jiankun He, Xinran Wang, Lijing Cai, Zhanli Jia, Chang Liu, Xuemei Sun, Si Wu, Chunyan Ding, Zi Zhang, Yueping Liu

**Affiliations:** Department of Pathology, the Fourth Hospital of Hebei Medical University, No. 12 Jiankang Road, Shijiazhuang, Hebei 050011 China

**Keywords:** Breast cancer, PD-L1, Immunohistochemistry, Paraffin sections, Storage time

## Abstract

**Background:**

PD-L1 staining using long-stored paraffin sections may not be consistent with the true PD-L1 expression of patients. Therefore, it is necessary to explore the expression of PD-L1(SP142) in paraffin sections of invasive breast cancer with different storage times and the optimal storage temperature for unstained paraffin sections.

**Methods:**

The study included 71 cases of PD-L1(SP142) positive breast cancer. The unstained paraffin sections were stored at room temperature conditions (20–25 °C), 4 °C, -20 °C and − 80 °C, respectively. PD-L1 staining was performed at 1, 2, 3, 4, 8, 12 and 24 weeks of storage. PD-L1 expression was assessed with a continuity score.

**Results:**

The PD-L1 antigen was gradually lost as the storage time of paraffin sections increased. The PD-L1 positivity rate was 97.18% at 1 week for the sections stored at room temperature, and decreased from 83.10 to 71.83% for the sections stored for 2 weeks to 4 weeks, and 61.97%, 54.93%, and 32.93% for 8, 12, and 24 weeks, respectively. When stored at low temperatures of 4 °C, -20 °C and − 80 °C, the positivity rate decreases with the same trend but more slowly compared to room temperature. The mean IC score of PD-L1 also showed a gradual decrease in all cases. In the consistency analysis, PD-L1 expression in slices stored at room temperature for 2 weeks was consistent with PD-L1 expression in fresh slices (ICC ≥ 0.9 for all slices), and PD-L1 expression in slices stored at 4 °C or -20 °C for 4 weeks was consistent with PD-L1 expression in fresh slices (ICC ≥ 0.9 for all slices). When stored under refrigeration at -80 °C, PD-L1 expression in slices stored for 3 weeks was consistent with that in fresh slices (ICC ≥ 0.9).

**Conclusions:**

To our knowledge, this is the first article on the effect of preservation time and preservation temperature of paraffin sections on PD-L1 expression in breast cancer. Long-term storage of paraffin sections of unstained invasive breast cancer can lead to antigen loss of PD-L1 (SP142). Refrigerated storage of paraffin sections can delay antigen loss, with best results at 4 °C or -20 °C, and a storage time of no more than 4 weeks is recommended.

**Supplementary Information:**

The online version contains supplementary material available at 10.1186/s13000-023-01423-8.

## Background

Programmed death-1 (PD-1) inhibits T cell function and prevents tumor cell destruction through interaction with programmed death-ligand 1 (PD-L1) [1, 2]. Immune checkpoint inhibitors that block PD-1/PD-L1 interactions have provided new therapeutic approaches for many malignancies [3–5]. PD-L1 inhibitors are one of the effective treatments for patients with unresectable locally advanced or metastatic triple-negative breast cancer. Several clinical trials, including IMpassion130, have demonstrated that the expression of PD-L1 (SP142) is the best predictor of the efficacy of atezolizumab in the treatment of locally advanced or metastatic triple-negative breast cancer compared to other antibodies [6–8]. Therefore PD-L1 (SP142) was approved by the US Food and Drug Administration as a concomitant diagnostic test for screening immunotherapy populations.PD-L1 inhibitor therapy is administered in such a way that only a small percentage of patients benefit, and some of these patients experience immune-related adverse events in the skin, gastrointestinal, hepatic, endocrine and respiratory systems [9]. Therefore accurate detection of PD-L1 would be beneficial for precise treatment of patients.

The results of immunohistochemistry (IHC) assays for biomarkers may be affected by a variety of factors. Earlier studies have shown that the use of long-stored paraffin sections for immunohistochemical staining can affect the accuracy of the test results [10, 11]. Prolonged storage of paraffin sections leads to loss of antibody antigenicity [12, 13]. Therefore antigenic immunoreactivity in paraffin sections is time-sensitive. In clinical practice and research, it is common for paraffin sections to be stored for long periods of time, which may result in false-negative results. The extent of antigenic loss depends on the tumor and the type of antigen, so it is necessary to explore PD-L1 expression in paraffin sections of invasive breast cancer with different storage times.

The degree of loss of antigenicity in paraffin sections can also be influenced by the conditions under which they are stored. Some studies suggest that the optimal storage conditions for unstained paraffin sections are not at room temperature [14–16]. Many options for storing paraffin slices have been proposed in studies, including vacuum storage, paraffin coating after slicing, storage in a nitrogen chamber, and addition of antioxidants [17]. However, many of the above experimental conditions require high laboratory hardware and are more difficult to implement in routine clinical work with high workloads. It is therefore necessary to find more stable and easier to implement storage solutions for paraffin sections.

Few articles have investigated the effects of preservation time and preservation temperature of paraffin sections on PD-L1 expression in breast cancer. Therefore, we aimed to explore PD-L1(SP142) expression in paraffin sections of invasive breast cancer stored for different times and the optimal storage conditions for paraffin sections. It is of significance for the precise diagnosis of invasive breast cancer patients and the quality control of PD-L1(SP142) IHC staining.

## Methods

### Patient cohort and data preparation

**Fig. 1:**
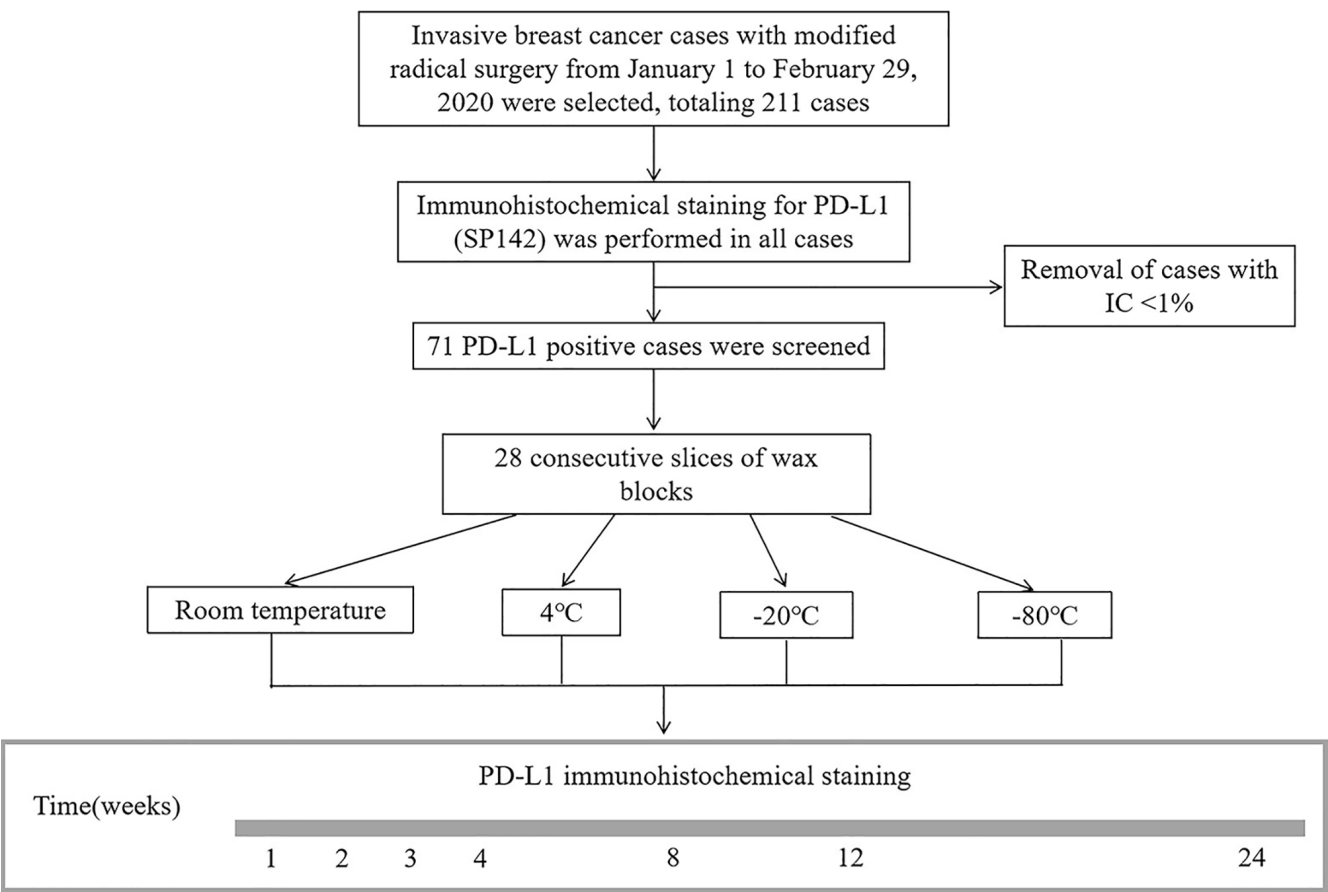
Schematic representation of study design

Invasive breast cancer cases that underwent modified radical surgery from January 1 to February 29, 2020 were selected, totaling 211 cases. All underwent immunohistochemical staining for PD-L1 (SP142) and 71 positive cases were screened. All 71 cases were serially sectioned in 28 slices, and unstained paraffin sections were stored at room temperature conditions (20–25 °C), 4 °C, -20 °C, and − 80 °C, respectively. PD-L1 (SP142) staining was performed at 1, 2, 3, 4, 8, 12 and 24 weeks of storage (Fig. 1).

### Immunohistochemistry and scoring

All sections stained with PD-L1 (clone SP142, Ventana Medical Systems, Tucson, USA) using the OptiView DAB IHC detection kit, strictly following the manufacturer’s instructions on benchmark XT automatic immunohistochemistry (IHC) (BenchMark ULTRA, Ventana, Tucson, USA).

PD-L1(SP142) antibody was scored using immune cells (IC) positivity score, defined as the percentage of PD-L1 immune cells with any intensity of staining within the tumor area. Immune cells were cells in the intratumor and adjacent peritumor stroma, including lymphocytes, macrophages, dendritic cells and granulocytes, excluding IC staining in the vascular cavity. Tumor area is defined as the area occupied by tumor cells and their associated intratumoral and periatumoral stroma, excluding necrotic area and carcinoma in situ area [8]. The continuity score was used to evaluate the expression of PD-L1. If the tumor interstitial infiltrating immune cells were positive ≥ 1%, the expression was considered positive. All sections were independently interpreted by two experienced pathologists. Discordant cases were reviewed under a multiocular microscope and consensus was reached by mutual consultation.

### Statistical analysis

Statistical analysis was performed using SPSS 23.0 and GraphPad Prism 8.0.1. Use two-way mixed consistency intragroup correlation coefficient (ICC) to evaluate the consistency of PD-L1 expression in paraffin sections at different times and conditions with fresh sections. The concordance was regarded as “poor”, “moderate”, “good” and “excellent” for the ICC values in (0,0.5), (0.5,0.75), (0.75, 0.9), and (0.9,1.0), respectively [18]. All tests were two-sided. Statistical significance was set at *p* < 0.05.

## Results

### Patient characteristics

A total of 71 patients were included in this study. The patients were all females with a mean age of 55 years (32–79 years). Histologic diagnosis was invasive ductal carcinoma in 67 cases (94.37%) and invasive lobular carcinoma in 4 cases (5.63%). Histologic grading was grade I in 2 cases, grade II in 49 cases, and grade III in 20 cases. There were 21 cases (29.58%) with tumor size less than 2 cm, 41 cases (57.75%) with tumor size between 2 and 3 cm, and 9 cases (12.67%) with tumor size greater than 3 cm. There were 42 cases of luminal type, 10 cases of HER-2 overexpression type, and 19 cases of triple-negative breast cancer. The clinical staging was 20 cases (28.17%) in stage I, 42 cases (59.15%) in stage II, 8 cases (11.27%) in stage III and 1 case (1.41%) in stage IV. PD-L1 was positively expressed in all cases. 43 cases (60.56%) had PD-L1 expression > 1 and ≤ 5, 16 cases (22.54%) had > 5 and ≤ 10, and 12 cases (16.9%) had > 10 (Supplementary Table 1).

### Changes in the positive rate of PD-L1 expression in paraffin sections at different storage temperatures at different times

**Fig. 2:**
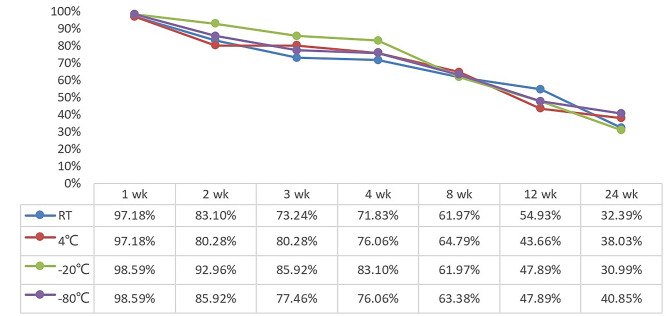
The positive staining rate of PD-L1 in sections at different storage temperatures and different time points The positive rate decreased gradually with the prolongation of storage time Note: wk:week RT: room temperature

**Fig. 3:**
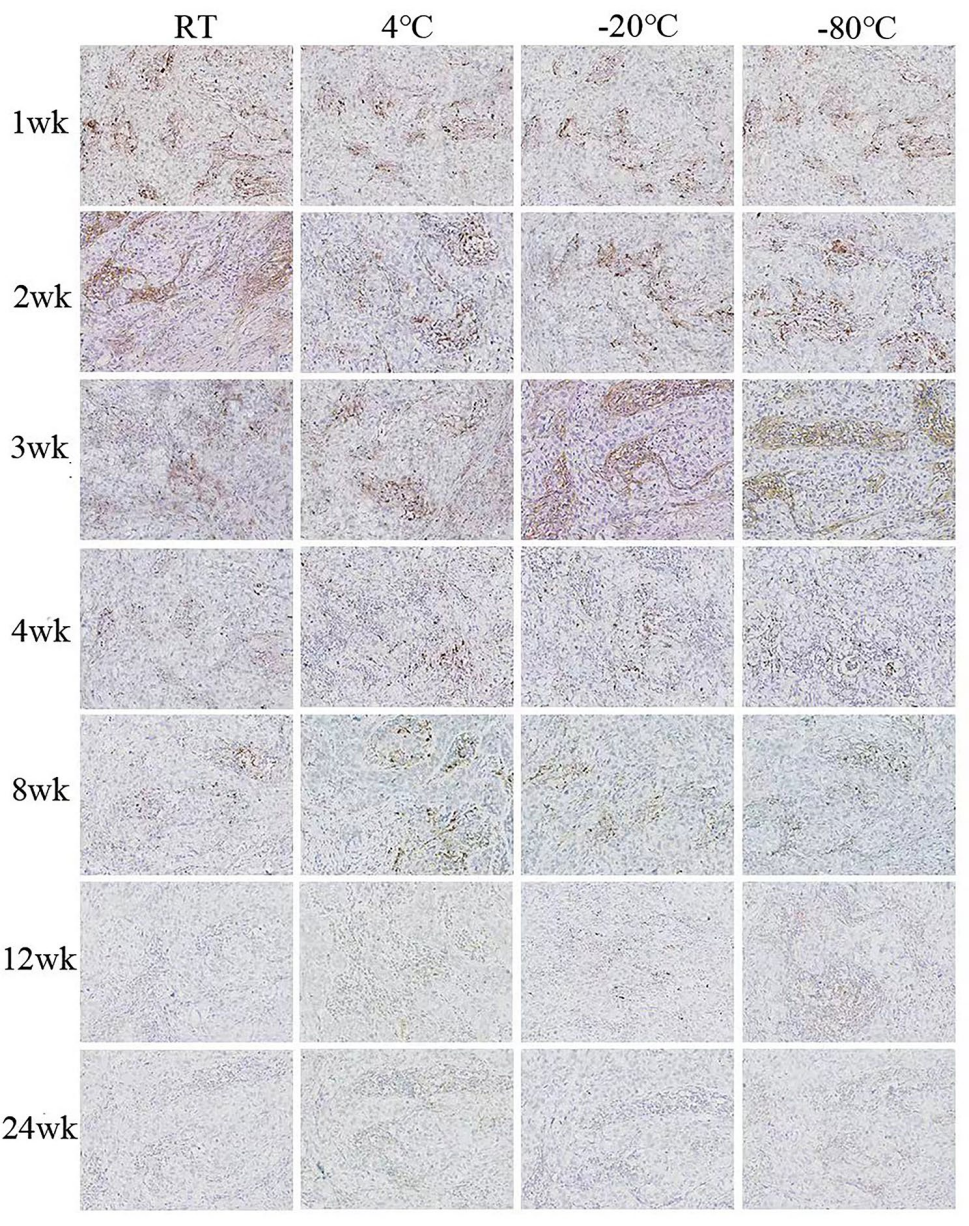
The expression of PD-L1 is different in breast cancer slices at different storage times at four storage temperatures(×100)

With the extension of storage time, the positivity rate of PD-L1 expression in paraffin sections gradually decreased (Fig. 2). A representative IHC example is shown in Fig. 3. The PD-L1 positivity rate for sections stored at room temperature was 97.18% at 1 week, decreased from 83.10 to 71.83% for sections stored from 2 weeks to 4 weeks, and was 61.97% when storage time reached 8 weeks. The PD-L1 positivity rate of the sections stored at 4 °C was 97.18% at 1 week, which decreased to 80.28% at 2 weeks, with no change at 3 weeks compared to 2 weeks, 76.06% at 4 weeks, and 64.79% at 8 weeks. The PD-L1 positivity rate of the slices stored at -20 °C was 98.59% at 1 week, decreased from 92.96 to 83.10% for the slices stored from 2 weeks to 4 weeks, and was higher than the other storage temperatures at each time point up to 4 weeks, with a positivity rate of 61.97% at 8 weeks. The PD-L1 positivity rate of slices stored at -80 °C was 98.59% at 1 week, decreased from 85.92 to 76.06% for slices stored from 2 weeks to 4 weeks, and was 63.38% at 8 weeks. The PD-L1 positivity rate at 12 weeks for sections stored at room temperature was 54.93%, and the PD-L1 positivity rate at 12 weeks for the rest of the sections stored at temperature was less than 50%.The PD-L1 positivity rate at 24 weeks of storage was about 35%.

### Changes in IC scores of PD-L1 expression in paraffin sections at different storage temperatures at different times

**Fig. 4:**
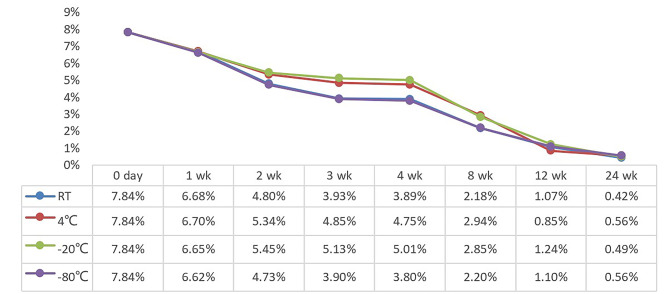
PD-L1 IC score of sections at different storage temperatures and different time points Note: wk:week RT: room temperature

The mean IC score of PD-L1 in paraffin sections also showed a gradual decrease with longer storage time. The mean IC score at the initial state of 71 slices was 7.84% (Fig. 4). At 1 week of storage, slice PD-L1 expression decreased by about 1% regardless of storage temperature. At 2 weeks of storage, the PD-L1 IC scores of sections stored at 4 °C and − 20 °C decreased less, and the average PD-L1 IC scores of sections stored at room temperature and − 80 °C were < 5%. At 3 and 4 weeks of storage, only the mean PD-L1 IC score of the sections stored at -20 °C was above 5%. After 8 weeks of storage, the mean IC score of PD-L1 of paraffin slices decreased more severely, below 3%, regardless of storage temperature.

### Comparison of the consistency of PD-L1 expression between paraffin sections and fresh sections at different storage temperatures and times

**Fig. 5:**
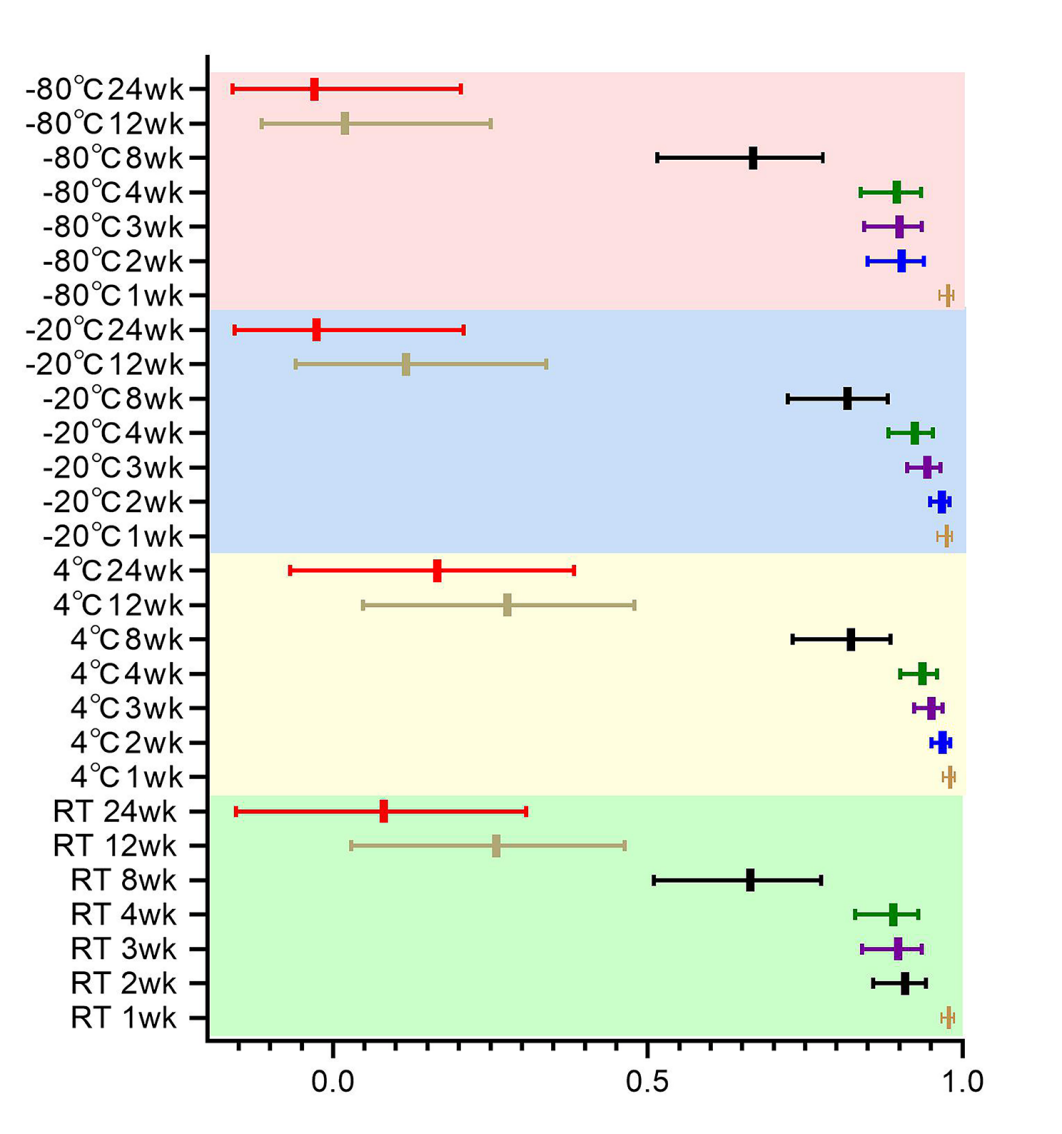
Comparison of the correlation between the expression of PD-L1 in sections at different time points and in fresh sections Note: wk:week RT: room temperature

To find the optimal storage temperature for unstained paraffin sections and to explore the longest storage timeframe in which PD-L1 staining could be performed, we compared the consistency of PD-L1 expression in sections stored at different temperatures with that in fresh sections at different time points (Fig. 5, Supplementary Table 2). For sections stored at room temperature, concordance of PD-L1 expression with fresh sections was excellent (ICC ≥ 0.9) for sections up to 2 weeks, good (ICC ≥ 0.75) at 4 weeks, and moderate (ICC ≥ 0.5) at 8 weeks. For paraffin sections stored at 4 °C and − 20 °C, the ICC of PD-L1 expression for sections stored up to 4 weeks compared with PD-L1 expression for fresh sections was > 0.9, and the concordance was evaluated as excellent. Concordance was good (ICC ≥ 0.75) for all sections at 8 weeks. -80 °C stored slices, PD-L1 expression in slices stored for up to 3 weeks showed excellent concordance with fresh slices (ICC ≥ 0.9), good concordance at 4 weeks (ICC ≥ 0.75), and moderate concordance at 8 weeks (ICC ≥ 0.5). When the preservation time reached 12 weeks, the PD-L1 expression of sections stored at each temperature was in poor concordance (ICC < 0.5) compared with fresh sections, and PD-L1 antigen loss was more severe. ICC < 0.2 at 24 weeks of storage does not reflect the true expression of PD-L1 in the slices.

## Discussion

Immunotherapy with PD-1/PD-L1 axis inhibitors is a revolution in the field of oncology and a new therapeutic approach to fight cancer [19]. PD-1 inhibits T cells, and drugs targeting PD-1 and PD-L1 have shown promising efficacy in the treatment of a number of highly immunogenic tumors (non-Hodgkin’s lymphoma, non-small cell lung cancer, and melanoma) [20, 21]. PD-L1 expression may vary at different disease sites and at different time points in the disease course [22]. Therefore, PD-L1 precise detection is of great significance for clinical diagnosis and treatment. It is common for unstained sections to be stored for long periods of time in clinical work, scientific research, and inter-room quality control of immunohistochemistry assays, but this may affect the expression of PD-L1 on the sections, leading to inaccurate final results. In this study in order to explore the PD-L1(SP142) staining of paraffin sections of invasive breast cancer stored for different times and the optimal storage conditions for paraffin sections. Consecutive paraffin sections from 71 patients with PD-L1(SP142) positive invasive breast cancer were included, and the paraffin sections were stored at room temperature (20–25 °C), 4 °C, -20 °C, and − 80 °C for different times for staining. The staining results were compared with PD-L1 expression in corresponding fresh sections. Continuous scoring of PD-L1 is better able to detect differences in antigen loss and has important implications for daily clinical practice.

Previous experiments have examined the effect of paraffin sections of breast cancer with different storage times on ER, PR, HER2 and Ki67 staining. This study showed that the immune responsiveness of tumor cells decreases as storage time increases [23]. However, the staining intensity of the estrogen receptor is not affected, and Vimentin even increase with storage time [24]. These studies suggest that the loss of antigenic immunoreactivity varies between antigens within and between tissue types. In this study, we found that PD-L1 expression in paraffin sections gradually decreased with the extension of storage time, regardless of the storage temperature. The PD-L1 positivity rate was 97.18% when the sections stored at room temperature were stored for 1 week, decreased from 83.10 to 71.83% for sections stored for 2 weeks to 4 weeks, and decreased considerably to 61.97% when the storage time reached 8 weeks. This is consistent with the trend of previous findings by Sato Y et al. and Alexander H et al. [25, 26]. The expression of PD-L1 decreased faster in our study. The reason may be that the high number of cases with low PD-L1 expression included in the experiment may also have some influence on the results. However, cases with critical values in clinical practice need to be screened more accurately, so paraffin sections should be stained for PD-L1 in a timely manner.

A study by Gelb et al. [27] showed that although fresh paraffin sections for PTEN staining are optimal, sections can be stored refrigerated for up to 2 months with or without paraffin coating and can be used for up to 4 months with paraffin coating. Omilian A R et al. [23] showed that storage in a desiccator is the most effective way to reduce immunoreactivity loss, paraffin-coated sections are technically more complex to store and have the greatest loss of immunoreactivity, and refrigeration at 4 °C is an intermediate option. Alexander H et al. showed that humidity and temperature have an important effect on antigen loss in FFPE tissues [26]. Considering the simplicity and feasibility of storage methods for paraffin sections in clinical practice, in this study, paraffin sections were stored at room temperature (20–25 °C), 4 °C, -20 °C, and − 80 °C to explore the protective effect of different storage temperatures on the antigenic immunoreactivity of paraffin sections. In this study, we found that PD-L1 expression in sections stored at 4 °C and − 20 °C for up to 4 weeks had an ICC of 0.9 or more compared with fresh sections, with excellent concordance in all cases, and good concordance in all cases at 8 weeks. For sections stored at room temperature, PD-L1 expression was in excellent concordance with fresh sections for sections up to 2 weeks, with good concordance at 4 weeks. -80 °C stored sections, the concordance of PD-L1 expression with fresh sections was excellent for sections up to 3 weeks, good at 4 weeks, and moderate at 8 weeks. When the preservation time reached 12 weeks, the PD-L1 expression of sections stored at each temperature was in poor concordance compared with fresh sections. ICC < 0.2 at 24 weeks of storage does not reflect the true expression of PD-L1 in the slices. Does not reflect the true level of PD-L1 expression in cases. According to our results, storing paraffin sections at 4 °C or -20 °C temperature reduced the loss of immunoreactivity of PD-L1.

The exact cause of antigen degradation has not been unanimously determined, and previous studies have shown that nuclear and cytoplasmic staining are less affected by storage time compared to cell membrane staining [13]. It has been shown that the expression of PD-L1 also decreases after the stained immunohistochemistry sections are stored for a certain period of time [28]. There are a variety of potential factors that have been suggested to influence antigen loss including: pre-analytical variables, oxidation, humidity and temperature [14, 16, 29]. Tissue handling conditions may interfere with immunohistochemical scoring, and staining conditions are an important influence on the quality of staining; therefore, strict adherence to specifications for tissue handling and fixation is required in all pathology facilities [30, 31]. In a study by Alexander H et al.paraffin sections of non-small cell lung cancer (NSCLC), gastric cancer, placenta, and tonsil tissues of varying storage durations were stained using four PD-L1 (22C3,28 − 8,E1L3N, and SP142) antibodies [26]. This study demonstrated that the expression of different PD-L1 antibody clones tended to decrease with prolonged storage time of paraffin sections, but not to the exact same extent, and thus the antibody clones should be given sufficient attention in clinical practice.That study concluded that humidity and high temperatures are the main causes of antigen loss, while oxidation is not.Our study also confirmed that paraffin sections stored at low degrees can reduce the loss of PD-L1 immunoreactivity, but the storage condition of -80 °C is not the best choice, so the mechanism of the effect of low temperature on antigen storage needs to be further investigated. Our study also has some limitations. First, we are a single-center study and included a limited sample size.We believe that further largescale studies are warranted to identify all possible candidates for therapy with PD-1 axis inhibitor and to establish optimal pathological processing methods for accurate PD-L1 assessment.Secondly there are limited methods for storage conditions of paraffin sections. It is expected that further research, using more storage methods, will find the specific reasons for the loss of antigen.

To our knowledge, this is the first article on the effect of preservation time and preservation temperature of paraffin sections on PD-L1 expression in breast cancer. Long-term storage of paraffin sections of unstained invasive breast cancer can lead to antigen loss of PD-L1 (SP142). Storage of paraffin sections under refrigeration delays antigen loss.Our study found that storage at 4 °C or -20 °C worked best. However, in order to more accurately screen patients who can undergo PD-L1 immunotherapy, it is recommended that unstained paraffin sections be stained for PD-L1 (SP142) within 4 weeks.

## Conclusions

Long-term storage of paraffin sections of unstained invasive breast cancer can lead to antigen loss of PD-L1 (SP142). Refrigerated storage of paraffin sections can delay antigen loss, with best results at 4 °C or -20 °C, and a storage time of no more than 4 weeks is recommended.

### Electronic supplementary material

Below is the link to the electronic supplementary material.

Supplementary Table 1: Clinicopathological Characteristics of patients

Supplementary Table 2: Consistency analysis of PD-L1 scores between sections stored at different temperatures and times and fresh sections

## Data Availability

All data generated or analysed during this study are included in this published article [and its supplementary information files].

## List of Abbreviations

BC: Breast Cancer
ER: Estrogen Receptors
PR: Progesterone Receptors
Ki67: Nuclcar-associated antigen Ki67
HER2: Human Epidermal Growth Factor Receptor 2
IHC: Immunohistochemistry
IDC: Invasive Ductal Carcinoma
ILC: Invasive Lobular Carcinoma
TNBC: Triple-Negative Breast Cancer
PD-L1: Programmed Cell Death-Ligand 1
PD-1: Programmed Death-1
ICC: Intraclass Correlation Coefficient
IC: Tumor-Infiltrating Immune Cell

## Declarations

### Animal Studies

N/A.

## References

[1] Butte M, Keir M, Phamduy T, Sharpe A, Freeman G (2007). Programmed death-1 ligand 1 interacts specifically with the B7-1 costimulatory molecule to inhibit T cell responses. Immunity.

[2] Wei S, Duffy C, Allison J (2018). Fundamental mechanisms of Immune Checkpoint Blockade Therapy. Cancer Discov.

[3] Ishida Y, Agata Y, Shibahara K, Honjo T (1992). Induced expression of PD-1, a novel member of the immunoglobulin gene superfamily, upon programmed cell death. EMBO J.

[4] Herbst R, Soria J, Kowanetz M, Fine G, Hamid O, Gordon M, Sosman J, McDermott D, Powderly J, Gettinger S (2014). Predictive correlates of response to the anti-PD-L1 antibody MPDL3280A in cancer patients. Nature.

[5] Akinleye A, Rasool Z (2019). Immune checkpoint inhibitors of PD-L1 as cancer therapeutics. J Hematol Oncol.

[6] Rugo HS, Loi S, Adams S, Schmid P, Schneeweiss A, Barrios CH, Iwata H, Diéras V, Winer EP, Kockx MM (2021). PD-L1 immunohistochemistry assay comparison in Atezolizumab plus nab-paclitaxel-treated Advanced Triple-negative Breast Cancer. JNCI-Journal of the National Cancer Institute.

[7] Adams S, Diéras V, Barrios C, Winer E, Schneeweiss A, Iwata H, Loi S, Patel S, Henschel V, Chui S (2020). Patient-reported outcomes from the phase III IMpassion130 trial of atezolizumab plus nab-paclitaxel in metastatic triple-negative Breast cancer. Annals of Oncology: Official Journal of the European Society for Medical Oncology.

[8] Schmid P, Adams S, Rugo H, Schneeweiss A, Barrios C, Iwata H, Diéras V, Hegg R, Im S, Shaw Wright G (2018). Atezolizumab and Nab-Paclitaxel in Advanced Triple-negative Breast Cancer. N Engl J Med.

[9] Postow M. Managing immune checkpoint-blocking antibody side effects. American Society of Clinical Oncology educational book American Society of Clinical Oncology Annual Meeting 2015:76–83. 10.14694/EdBook_AM.2015.35.76 25993145

[10] Wester K, Wahlund E, Sundström C, Ranefall P, Bengtsson E, Russell P, Ow K, Malmström P, Busch C (2000). Paraffin section storage and immunohistochemistry. Effects of time, temperature, fixation, and retrieval protocol with emphasis on p53 protein and MIB1 antigen. Appl Immunohistochem Mol Morphology: AIMM.

[11] DiVito K, Charette L, Rimm D, Camp R (2004). Long-term preservation of antigenicity on tissue microarrays. Lab Invest.

[12] Jacobs T, Prioleau J, Stillman I, Schnitt S (1996). Loss of Tumor marker-immunostaining intensity on stored paraffin slides of Breast cancer. J Natl Cancer Inst.

[13] van den Broek L, van de Vijver M (2000). Assessment of problems in diagnostic and research immunohistochemistry associated with epitope instability in stored paraffin sections. Appl Immunohistochem Mol Morphology: AIMM.

[14] Economou M, Schöni L, Hammer C, Galván J, Mueller D, Zlobec I (2014). Proper paraffin slide storage is crucial for translational research projects involving immunohistochemistry stains. Clin Translational Med.

[15] Fergenbaum J, Garcia-Closas M, Hewitt S, Lissowska J, Sakoda L, Sherman M. Loss of antigenicity in stored sections of Breast cancer tissue microarrays. Cancer epidemiology, biomarkers & prevention: a publication of the American Association for Cancer Research, cosponsored by the American Society of Preventive Oncology 2004, 13(4):667–72. 15066936

[16] Grillo F, Pigozzi S, Ceriolo P, Calamaro P, Fiocca R, Mastracci L (2015). Factors affecting immunoreactivity in long-term storage of formalin-fixed paraffin-embedded tissue sections. Histochem Cell Biol.

[17] Engel K, Moore H (2011). Effects of preanalytical variables on the detection of proteins by immunohistochemistry in formalin-fixed, paraffin-embedded tissue. Arch Pathol Lab Med.

[18] Koo T, Li M (2016). A Guideline of selecting and reporting Intraclass correlation coefficients for Reliability Research. J Chiropr Med.

[19] Brahmer J, Pardoll D (2013). Immune checkpoint inhibitors: making immunotherapy a reality for the treatment of Lung cancer. Cancer Immunol Res.

[20] Konishi J, Yamazaki K, Azuma M, Kinoshita I, Dosaka-Akita H, Nishimura M (2004). B7-H1 expression on non-small cell Lung cancer cells and its relationship with tumor-infiltrating lymphocytes and their PD-1 expression. Clin cancer Research: Official J Am Association Cancer Res.

[21] Hino R, Kabashima K, Kato Y, Yagi H, Nakamura M, Honjo T, Okazaki T, Tokura Y (2010). Tumor cell expression of programmed cell death-1 ligand 1 is a prognostic factor for malignant Melanoma. Cancer.

[22] Hong L, Negrao M, Dibaj S, Chen R, Reuben A, Bohac J, Liu X, Skoulidis F, Gay C, Cascone T (2020). Programmed death-ligand 1 heterogeneity and its impact on Benefit from Immune checkpoint inhibitors in NSCLC. J Thorac Oncology: Official Publication Int Association Study Lung Cancer.

[23] Omilian A, Zirpoli G, Cheng T, Yao S, Stein L, Davis W, Head K, Nair P, Khoury T, Ambrosone C et al. Storage Conditions and Immunoreactivity of Breast Cancer Subtyping Markers in Tissue Microarray Sections. Applied immunohistochemistry & molecular morphology: AIMM 2020, 28(4):267–273. 10.1097/PAI.0000000000000756 PMC6906262 31205070

[24] Bertheau P, Cazals-Hatem D, Meignin V, de Roquancourt A, Vérola O, Lesourd A, Séné C, Brocheriou C, Janin A (1998). Variability of immunohistochemical reactivity on stored paraffin slides. J Clin Pathol.

[25] Sato Y, Fujimoto D, Uehara K, Kawachi H, Nagata K, Nakagawa A, Otsuka K, Sakanoue I, Hamakawa H, Takahashi Y (2018). Reduced Tumour proportion scores for programmed cell death Ligand 1 in stored paraffin tissue sections. Anticancer Res.

[26] Haragan A, Liebler DC, Das DM, Soper MD, Morrison RD, Slebos RJC, Ackermann BL, Fill JA, Schade AE, Gosney JR (2020). Accelerated instability testing reveals quantitative mass spectrometry overcomes specimen storage limitations associated with PD-L1 immunohistochemistry. Lab Invest.

[27] Gelb A, Freeman V, Astrow S (2011). Evaluation of methods for preserving PTEN antigenicity in stored paraffin sections. Appl Immunohistochem Mol Morphology: AIMM.

[28] Karpathiou G, Vincent M, Dumollard JM, Mobarki M, Péoc’h M (2022). PD-L1 expression in Head and Neck cancer tissue specimens decreases with time. Pathol Res Pract.

[29] Ramos-Vara JA, Webster JD, DuSold D, Miller MA (2014). Immunohistochemical evaluation of the effects of paraffin section storage on biomarker stability. Vet Pathol.

[30] Allison K, Hammond M, Dowsett M, McKernin S, Carey L, Fitzgibbons P, Hayes D, Lakhani S, Chavez-MacGregor M, Perlmutter J (2020). Estrogen and progesterone receptor testing in Breast Cancer: ASCO/CAP Guideline Update. J Clin Oncology: Official J Am Soc Clin Oncol.

[31] Leung S, Nielsen T, Zabaglo L, Arun I, Badve S, Bane A, Bartlett J, Borgquist S, Chang M, Dodson A (2019). Analytical validation of a standardised scoring protocol for Ki67 immunohistochemistry on Breast cancer excision whole sections: an international multicentre collaboration. Histopathology.

