# Supplementary material for: Effect of storage time of paraffin sections on the expression of PD-L1 (SP142) in invasive breast cancer
Source: Diagn Pathol. 2023 Dec 5;18:131. doi: 10.1186/s13000-023-01423-8 (PMC10696808; doi:10.1186/s13000-023-01423-8)
Supplement: Supplementary file 1 — Supplementary Table 1: Clinicopathological Characteristics of patients [file 13000_2023_1423_MOESM1_ESM.docx]

Supplementary Table 1 Clinicopathological Characteristics of patients

| Characteristic | | Case（N＝71） |
| --- | --- | --- |
| Age | ≤55years | 35（49.30%） |
|  | ＞55years | 36（50.70%） |
| Histological type | IDC | 67（94.37%） |
|  | ILC | 4（5.63%） |
| Histological grade | Ⅰ | 2（2.82%） |
|  | Ⅱ | 49（69.01%） |
|  | Ⅲ | 20（28.17%） |
| Tumor size | ＜2cm | 21（29.58%） |
|  | 2cm-3cm | 41（57.75%） |
|  | ＞3cm | 9（12.67%） |
| Molecular subtype | Luminal | 42（59.16%） |
|  | HER2-positive | 10（14.08%） |
|  | TNBC | 19（26.76%） |
| TNM stage | Ⅰ | 20（28.17%） |
|  | Ⅱ | 42（59.15%） |
|  | Ⅲ | 8（11.27%） |
|  | Ⅳ | 1（1.41%） |
| PD-L1 | ＞1 and ≤5 | 43（60.56%） |
|  | ＞5 and ≤10 | 16（22.54%） |
|  | ＞10 | 12（16.90%） |
