# Supplementary material for: Effect of storage time of paraffin sections on the expression of PD-L1 (SP142) in invasive breast cancer
Source: Diagn Pathol. 2023 Dec 5;18:131. doi: 10.1186/s13000-023-01423-8 (PMC10696808; doi:10.1186/s13000-023-01423-8)
Supplement: Supplementary file 2 — Supplementary Table 2: Consistency analysis of PD-L1 scores between sections stored at different temperatures and times and fresh sections [file 13000_2023_1423_MOESM2_ESM.docx]

Supplementary Table 2 Consistency analysis of PD-L1 scores between sections stored at different temperatures and times and fresh sections

| Storage Temperature | Duration of Storage | ICC | 95% CI | Reliability evaluation |
| --- | --- | --- | --- | --- |
| room temperature | 1 wk | 0.979 | 0.967-0.987 | excellent |
|  | 2 wk | 0.909 | 0.858-0.942 | excellent |
|  | 3 wk | 0.898 | 0.841-0.935 | good |
|  | 4 wk | 0.890 | 0.830-0.930 | good |
|  | 8 wk | 0.663 | 0.509-0.775 | moderate |
|  | 12 wk | 0.259 | 0.029-0.463 | poor |
|  | 24 wk | 0.081 | -0.154-0.307 | poor |
| 4℃ | 1 wk | 0.981 | 0.969-0.988 | excellent |
|  | 2 wk | 0.969 | 0.951-0.981 | excellent |
|  | 3 wk | 0.951 | 0.923-0.969 | excellent |
|  | 4 wk | 0.937 | 0.901-0.960 | excellent |
|  | 8 wk | 0.823 | 0.730-0.886 | good |
|  | 12 wk | 0.277 | 0.048-0.478 | poor |
|  | 24 wk | 0.166 | -0.068-0.383 | poor |
| -20℃ | 1 wk | 0.975 | 0.960-0.984 | excellent |
|  | 2 wk | 0.968 | 0.949-0.980 | excellent |
|  | 3 wk | 0.944 | 0.912-0.965 | excellent |
|  | 4 wk | 0.925 | 0.883-0.953 | excellent |
|  | 8 wk | 0.817 | 0.722-0.882 | good |
|  | 12 wk | 0.116 | -0.119-0.339 | poor |
|  | 24 wk | -0.026 | -0.256-0.207 | poor |
| -80℃ | 1 wk | 0.977 | 0.964-0.986 | excellent |
|  | 2 wk | 0.904 | 0.850-0.939 | excellent |
|  | 3 wk | 0.900 | 0.844-0.936 | excellent |
|  | 4 wk | 0.896 | 0.839-0.934 | good |
|  | 8 wk | 0.667 | 0.515-0.778 | moderate |
|  | 12 wk | 0.019 | -0.214-0.250 | poor |
|  | 24 wk | -0.030 | -0.260-0.203 | poor |
